# Ionizing radiation affects the expression of Toll-like receptors 2 and 4 in human monocytic cells through c-Jun N-terminal kinase activation

**DOI:** 10.1093/jrr/rru040

**Published:** 2014-06-13

**Authors:** Hironori Yoshino, Kanae Chiba, Takahiro Saitoh, Ikuo Kashiwakura

**Affiliations:** Department of Radiological Life Sciences, Division of Medical Life Sciences, Hirosaki University Graduate School of Health Sciences, 66–1 Hon-cho, Hirosaki, Aomori 036–8564, Japan

**Keywords:** Toll-like receptor 2, Toll-like receptor 4, ionizing radiation, c-Jun N-terminal kinase

## Abstract

Pattern recognition receptors recognize pathogen-associated molecular patterns. Among these, Toll-like receptors (TLRs) have well-characterized roles in antibacterial and antiviral immunity. In the present study, the effects of ionizing radiation on the expression of TLRs and cellular responses to ligands were investigated in THP1 monocytes (human monocytic leukemia cells) and THP1-derived macrophage cells (macrophage-like cells), which are induced by culturing in the presence of phorbol 12-myristate 13-acetate. TLR2 and TLR4 expression was detected in THP1 and macrophage-like cells. X-irradiation caused increased expression of these TLRs in THP1 and decreased expression in macrophage-like cells. Responses to FSL-1 (TLR2 ligand) and lipopolysaccharide (LPS, TLR4 ligand) were estimated by determining the induction of tumor necrosis factor-α (TNF-α). After FSL-1 or LPS stimulation, TNF-α induction was greater in X-irradiated THP1 monocytes than in non-irradiated cells. However, although TNF-α expression was not affected by X-irradiation in macrophage-like cells, the expression of LPS-inducible interferon-β was lower following X-irradiation of macrophage-like cells. To clarify the mechanisms of TLR2 and TLR4 regulation by X-irradiation, expression of mitogen-activated protein kinase was investigated. These experiments showed that c-Jun N-terminal kinase (JNK) mediated increases in TLR expression in X-irradiated THP1 monocytes and decreases in TLR expression in X-irradiated macrophage-like cells. This study demonstrates that ionizing radiation modulates ligand-responsive TLR expression through the JNK pathway, depending on differentiation state.

## INTRODUCTION

Adaptive immunity involves antigen-specific immune responses of T and B cells, whereas innate immunity is mediated by antigen-presenting cells, such as macrophages and dendritic cells (DCs), that initiate adaptive immune responses following pathogen recognition [1, 2]. The innate immune system recognizes pathogen-associated molecular patterns (PAMPs) through pattern recognition receptors (PRRs). To date, several PRRs have been identified, including Toll-like receptors (TLRs), retinoic acid-inducible gene-I-like receptors and nucleotide-binding oligomerization domain-like receptors [3–6].

TLRs are well-studied PRRs that are indispensable for antibacterial and antiviral immunity, and 12 members of the TLR family have been identified in mammals [7, 8]. TLRs are receptive to various components of bacterial cell walls. For example, TLR2 and TLR4 recognize peptidoglycan from gram-negative bacteria and lipopolysaccharide (LPS) from gram-positive bacteria, and subsequently initiate host defense responses against bacteria. In contrast, TLR3 and TLR9 recognize genes of single-strand RNA viruses and DNA viruses such as herpes simplex virus, and initiate the production of antiviral cytokines such as type I interferon (IFN).

Both T and B cells of the adaptive immune system are highly sensitive to ionizing radiation, whereas antigen-presenting cells such as macrophages and DCs are relatively resistant to ionizing radiation-induced cell death, although radiation-induced functional impairments have been reported [9–11]. We previously showed that X-irradiated human monocytes, which are the precursors of DCs, could differentiate into DCs [12]. Furthermore, although DCs derived from X-irradiated monocytes showed low cell surface antigen expression and cytokine responses, and impaired ability to stimulate T cells following exposure to LPS, these cells remained responsive to the mixture of pro-inflammatory cytokines after X-irradiation [13]. These observations suggest that X-irradiation affects TLR expression and responsiveness to ligands. However, few studies have assessed the effects of X-irradiation on TLRs, despite the importance of understanding the effects of ionizing radiation on innate immunity. In the present study, we investigated the effects of ionizing radiation on the expression and ligand responses of TLR2 and TLR4 in THP1 human monocytic cells and THP1-derived macrophages.

## MATERIALS AND METHODS

### Reagents

LPS (*Escherichia coli* 055:B5) and phorbol 12-myristate 13-acetate (PMA) were purchased from Sigma-Aldrich (St Louis, MO, USA). The TLR2/TLR6 agonist FSL-1 was purchased from InvivoGen (San Diego, CA, USA). The fluorescence-labeled monoclonal antibodies (mAbs), anti-human TLR2-phycoerythrin (TLR2-PE) and TLR4-PE were purchased from eBioscience (San Diego, CA, USA). Anti-human tumor necrosis factor-α-fluorescein isothiocyanate (TNF-α-FITC) and mouse IgG_2a_-PE were purchased from Becton Dickinson (San Jose, CA, USA). Mouse IgG_1_-FITC was purchased from Beckman Coulter (Fullerton, CA, USA). Phospho-SAPK/c-Jun N-terminal kinase (JNK) (Thr183/Tyr185) mouse mAb, phospho-p38 mitogen-activated protein kinase (MAPK; Thr180/Tyr182) rabbit mAb, phospho-p44/42 MAPK [Extracellular signal-regulated kinase (ERK)1/2; Thr202/Tyr204] rabbit mAb, and Alexa Fluor® 488-conjugated goat anti-rabbit IgG were purchased from Cell Signaling Technology Japan, KK (Tokyo, Japan). Alexa Fluor® 488-conjugated goat anti-mouse IgG was purchased from Invitrogen (Carlsbad, CA, USA). SP600125 and PD98059 were purchased from Sigma-Aldrich, and SB203580 was obtained from Calbiochem (San Diego, CA, USA).

### Cell culture

THP1 human acute monocytic leukemia cells were obtained from RIKEN Bio-Resource Center (Tsukuba, Japan). Cells were cultured in RPMI1640 supplemented with 1% penicillin streptomycin (Gibco, Grand Island, NY, USA) and 10% heat-inactivated fetal bovine serum (FBS; Japan Bioserum Co. Ltd, Japan) at 37°C in a humidified atmosphere containing 5% CO_2_.

**Fig. 1.**
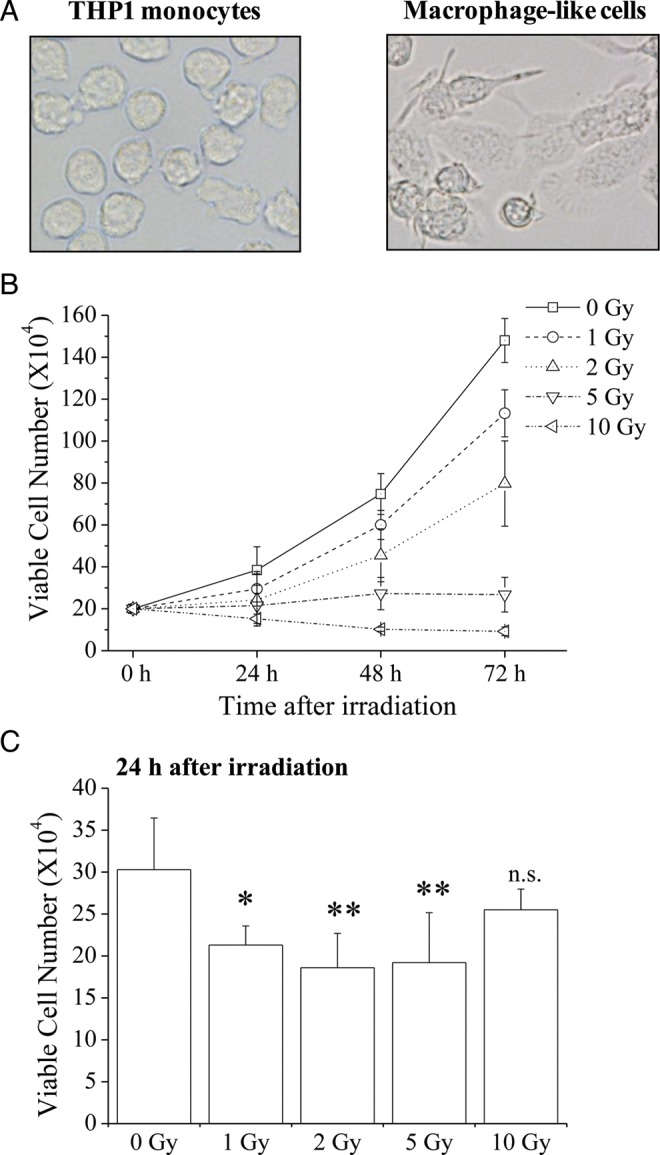
Cell morphology and viable cell numbers of X-irradiated THP1 monocytes and macrophage-like cells. (**A**) Morphology of THP1 monocytes (left) and macrophage-like cells (right). (**B**) THP1 monocytes were exposed to X-irradiation and were cultured for 24–72 h. Cells were harvested and viable cell numbers were estimated using the trypan blue dye exclusion method. (**C**) Macrophage-like cells were exposed to X-irradiation and were cultured for 24 h. Cells were harvested and viable cell numbers were estimated using the trypan blue dye exclusion method. Data are presented as the mean ± SD of four independent experiments; single and double asterisks indicate *P* < 0.05 and *P* < 0.01 compared with non-irradiated control, respectively; n.s. = not significant.

THP1-derived macrophages (macrophage-like cells) were prepared as previously described [14]. Briefly, THP1 monocytes (2.0 × 10^5^ cells/ml) were plated in 60 mm dishes (IWAKI, Tokyo, Japan) with 4 ml of medium containing 100 ng/ml PMA, and were cultured for 48 h. Differentiation to macrophage-like cells was confirmed by observing morphological changes under a microscope, as shown in Fig. 1A. After 48-h culture, the medium containing PMA was replaced with fresh medium not containing PMA, and macrophage-like cells were then used in experiments. Adherent macrophage-like cells were harvested by trypsinization with 0.1% trypsin/ethylenediaminetetraacetate (Gibco). Viable cell numbers were estimated using trypan blue dye exclusion assays.

### *In vitro* X-irradiation

X-irradiation (150 kVp, 20 mA, 0.5 mm Al, and 0.3 mm Cu filters) was performed using an X-ray generator (MBR-1520R-3; Hitachi Medical Corporation, Tokyo, Japan) at a distance of 45 cm from the focus and a dose rate of 1.00–1.04 Gy/min.

### Cell surface staining

THP1 monocytes (1.0 × 10^5^ cells/ml) or macrophage-like cells (about 2.0 × 10^5^ cells/ml) were exposed to X-rays and were harvested after 24 h for surface marker analyses. Cells were stained with TLR2-PE or TLR4-PE mAbs for 30 min at 4°C in the dark. Cells were also stained with corresponding PE-conjugated isotype control mouse IgG. After 30 min, the cells were washed with cold PBS (−) and were analyzed using a flow cytometer (Cytomics FC500; Beckman Coulter).

In experiments with MAPK inhibitors, 20 μM PD98059 (ERK inhibitor), SB203580 (p38 inhibitor) or SP600125 (JNK inhibitor) were added to the culture medium 30 min before X-irradiation.

### Measurements of TNF-α concentrations in culture supernatants

THP1 monocytes and macrophage-like cells were exposed to X-rays, and then LPS (1 μg/ml) or FSL-1 (50 ng/ml) were added at 24 h after X-irradiation. After further culture for 24 h, culture supernatants were collected for TNF-α measurements using a Quantikine Human TNF-α ELISA Kit (R&D Systems, Minneapolis, MN, USA) according to the manufacturer's instructions. The lowest detectable concentration was 15.6 pg/ml.

### Intracellular TNF-α staining

Intracellular TNF-α expression was analyzed using a Fixation and Permeabilization Solution Kit with BD GolgiPlug^TM^ (BD Bioscience). Briefly, THP1 monocytes (1 × 10^5^ cells/ml) or macrophage-like cells (about 2.0 × 10^5^ cells/ml) were exposed to X-rays. At 24 h after X-irradiation, LPS (1 μg/ml) or FSL-1 (50 ng/ml) was added to cultures, and the cells were cultured for an additional 8 h. GolgiPlug, which inhibits protein transporters, was added to the culture medium after 2 h of an 8-h culture. Cells were harvested and fixed using Cytofix/Cytoperm^TM^ solution (BD Bioscience) for 15 min on ice. After washing twice in Perm/Wash^TM^ solution (BD Bioscience), cells were suspended in Perm/Wash^TM^ solution and were stained with anti-human TNF-α-FITC mAbs or isotype control at room temperature in the dark. After 30 min, the cells were washed with Perm/Wash^TM^ solution and were analyzed using a flow cytometer.

### Intracellular phosphoprotein staining

Intracellular phosphorylated MAPK expression was analyzed using a flow cytometer. Briefly, THP1 monocytes (1 × 10^5^ cells/ml) or macrophage-like cells (about 2.0 × 10^5^ cells/ml) were exposed to X-rays. At 1 or 3 h after X-irradiation, cell suspensions were mixed with pre-warmed BD Cytofix buffer (BD Biosciences) and were incubated at 37°C in a water bath for 10 min. After washing with Washing/Staining Solution (PBS containing 1% FBS and 0.09% sodium azide), cells were permeabilized with cold Perm Buffer III (BD Biosciences) for 30 min on ice. After washing twice with Washing/Staining Solution, cells were suspended in Washing/Staining Solution and were labeled with primary phosphorylated MAPK (p-ERK, p-JNK and p-p38) antibodies for 30 min at room temperature. Labeled cells were washed twice with Washing/Staining Solution and were then stained with Alexa Fluor® 488-conjugated anti-mouse or Alexa Fluor® 488-conjugated anti-goat secondary antibodies at room temperature in the dark. As an isotype control, cells were stained with Alexa Fluor® 488-conjugated secondary antibodies alone. After 30 min, cells were washed with Washing/Staining Solution and were analyzed using flow cytometry.

### Reverse transcription polymerase chain reaction

Macrophage-like cells were exposed to X-rays, and LPS (1 μg/ml) was added to the culture at 24 h after X-irradiation. After an additional 24 h, cells were harvested and total RNA was extracted using an RNeasy MINI kit (Qiagen, Valencia, CA, USA). Total RNA was quantified using a NanoDrop (Thermo, Wilmington, DE, USA) and cDNA templates were synthesized from 1 μg RNA using oligo (dT)_12–18_ primers and SuperScript^TM^ III Reverse Transcriptase (Invitrogen), according to the manufacturer's instructions. Primers for IFN-β were 5′-CTT GTG GCA ATT GAA TGG GAG GC-3′ (sense) and 5′-CCA GGC ACA GTG ACT GTA CTC CTT-3′ (antisense). β-actin primers were 5′-GGC ACC CAG CAC AAT GAA GA-3′ and 5′-GGC ACG AAG GCT CAT CAT TC-3′ (antisense). PCR was performed using an AccuPrime^TM^ Taq DNA Polymerase System (Invitrogen). The reaction conditions for IFN-β were 94°C for 1 min followed by 35 cycles of 95°C for 30 s, 55°C for 2 min, and 72°C for 2 min, and then 72°C for 10 min. The reaction conditions for β-actin were 94°C for 2 min flowed by 18 cycles of 94°C for 30 s, 60°C for 30 s, and 72°C for 1 min, and then 72°C for 10 min. PCR products were confirmed using electrophoresis on ethidium bromide-stained 1.5% agarose gels. The sizes of the products for IFN-β and β-actin were 370 and 632 bp, respectively.

### Real-time quantitative reverse transcription polymerase chain reaction

Real-time quantitative reverse transcription polymerase chain reaction (RT-PCR) was performed using Power SYBR® Green (Applied Biosystems Inc., Carlsbad, CA, USA) and a StepOnePlus^TM^ system (Applied Biosystems Inc.) with typical amplification parameters (95°C for 10 min, followed by 40 cycles of 95°C for 15 s and 60°C for 1 min). Differences in gene expression relative to non-irradiated controls were determined using ΔCt values after normalization to the housekeeping gene β-actin. β-actin primer sequences are reported elsewhere [15]. Primers for IFN-β were 5′-GAT TCA TCT AGC ACT GGC TGG-3′ (sense) and 5′-CTT CAG GTA ATG CAG AAT CC-3′ (antisense).

### Statistical analysis

Data are presented as the mean ± SD. Comparisons of non-irradiated and X-irradiated groups were made using a one-way ANOVA model and the Dunnett's test or Steel's test, depending on the normality of data distributions. Differences were considered significant when *P* < 0.05. Statistical analyses were performed using Excel 2010 software (Microsoft, USA) with the add-in software Statcel 3 [16].

## RESULTS

### X-irradiation decreases numbers of viable THP1 monocytes and macrophage-like cells

The effects of ionizing radiation on viable cell numbers of THP1 monocytes and macrophage-like cells were investigated using trypan blue dye exclusion assays. THP1 monocytes retained the ability to grow after exposure to 1 or 2 Gy; however, viable cell numbers were decreased compared with non-irradiated cells (Fig. 1B), and cell growth was not observed after exposure to 5 or 10 Gy. Although macrophage-like cells did not grow after differentiation, and no significant differences in viable cell numbers were observed between non-irradiated and 10 Gy-irradiated cells, cells exposed to lower X-ray doses were significantly fewer than non-irradiated cells (Fig. 1C).

### X-irradiation upregulates cell surface TLR expression in THP1 monocytes and enhances responses to ligands

**Fig. 2.**
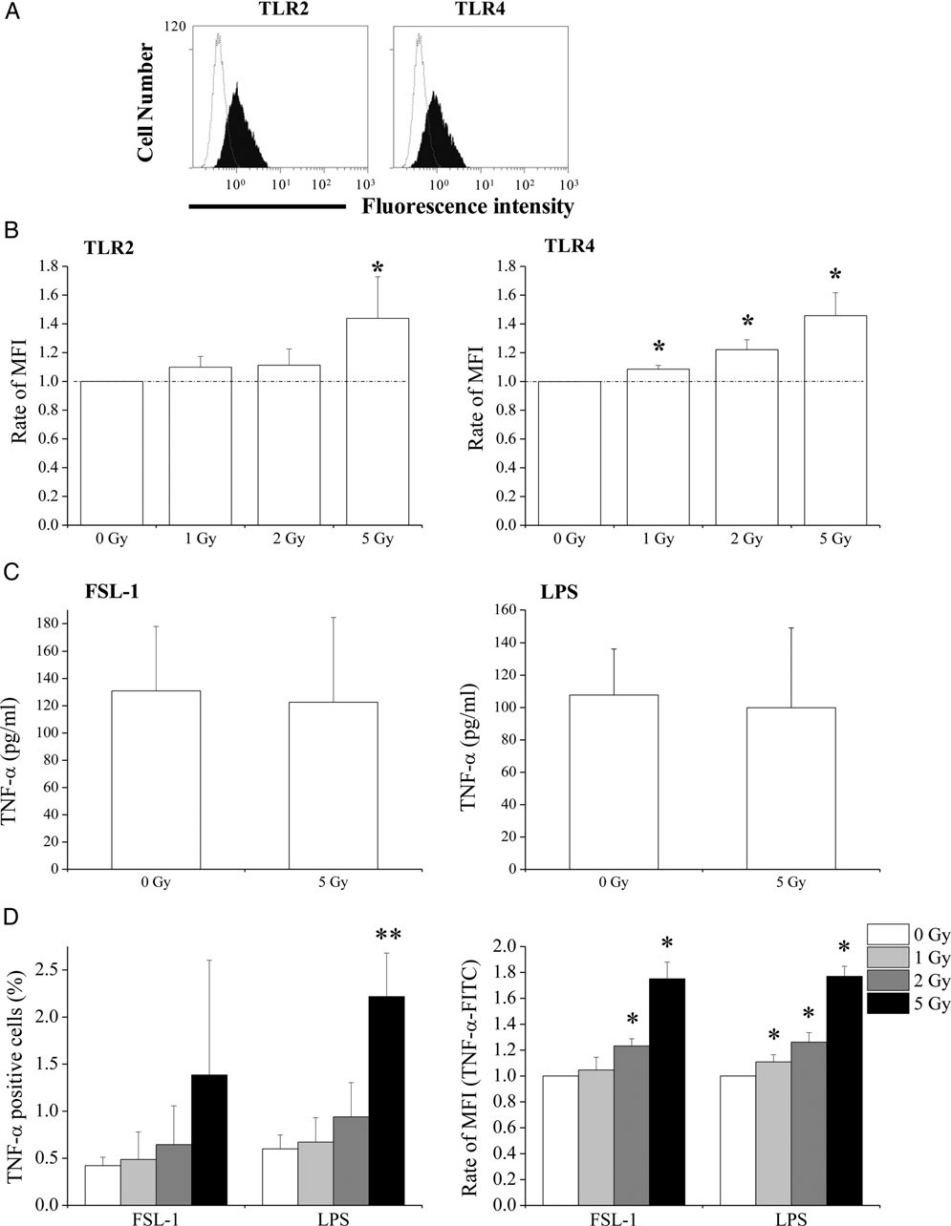
Effects of X-irradiation on TLR2 and TLR4 expression, and THP1 monocytes responses to ligands. (**A**) Representative histograms of TLR2 and TLR4 expression in non-irradiated THP1 monocytes; the dotted line indicates the isotype control. (**B**) Non- or X-irradiated THP1 monocytes were cultured for 24 h, and TLR2 and TLR4 expression was analyzed using flow cytometry; MFI = mean fluorescence intensity. Data are presented as the mean ± SD of four independent experiments. (**C**) Non- or 5 Gy-irradiated THP1 monocytes were cultured for 24 h, and FSL-1 or LPS were added to culture supernatants. After an additional 24 h of culture, supernatants were harvested and TNF-α concentrations were determined using ELISA. Data are presented as the mean ± SD of four independent experiments. (**D**) Non- or X-irradiated THP1 monocytes were cultured for 24 h. Cells were then stimulated with FSL-1 or LPS for 8 h, and the expression of intracellular TNF-α was determined. Data are presented as the mean ± SD of four independent experiments; single and double asterisks indicate *P* < 0.05 and *P* < 0.01 compared with non-irradiated control, respectively.

The effect of X-irradiation on TLR2 and TLR4 expression in THP1 monocytes was investigated. As shown in Fig. 2A, non-irradiated THP1 expressed both TLR2 and TLR4 on the cell surface. After 24-h exposure of THP1 monocytes to 5 Gy, the expression of TLR2 was increased (Fig. 2B). Similarly, the expression of TLR4 on X-irradiated THP1 monocytes was significantly higher than in non-irradiated cells. Because X-irradiation with 10 Gy dramatically decreased THP1 cell numbers, TLR expression was not analyzed in these cells.

X-irradiation with 5 Gy increased the expression of TLR2 and TLR4. Thus, the effect of X-irradiation on receptor responses to ligands was estimated according to the production of the pro-inflammatory cytokine TNF-α. Although culture supernatants from non-stimulated THP1 monocytes did not contain detectable TNF-α ( < 15.6 pg/ml), TNF-α was detectable after stimulation with FSL-1 (ligand for TLR2) or LPS (ligand for TLR4) for 24 h (Fig. 2C). No differences in TNF-α concentrations after stimulation with FSL-1 or LPS were observed between non-irradiated and 5 Gy-irradiated THP1 monocytes (Fig. 2C). However, given that viable cells decreased to 50% and 75% at 24 h and 48 h, respectively, after X-irradiation with 5 Gy (Fig. 1B), the production of TNF-α probably increased at least 2-fold in 5 Gy-irradiated THP1 monocytes compared with non-irradiated cells. Accordingly, analyses of intracellular TNF-α showed increases in the percentage of TNF-α-FITC-positive cells and in the mean fluorescence intensity of TNF-α-FITC after stimulation of 5 Gy-irradiated cells with FSL-1 or LPS compared with non-irradiated cells (Fig. 2D).

### X-irradiation downregulates cell surface TLR expression in macrophage-like cells

**Fig. 3.**
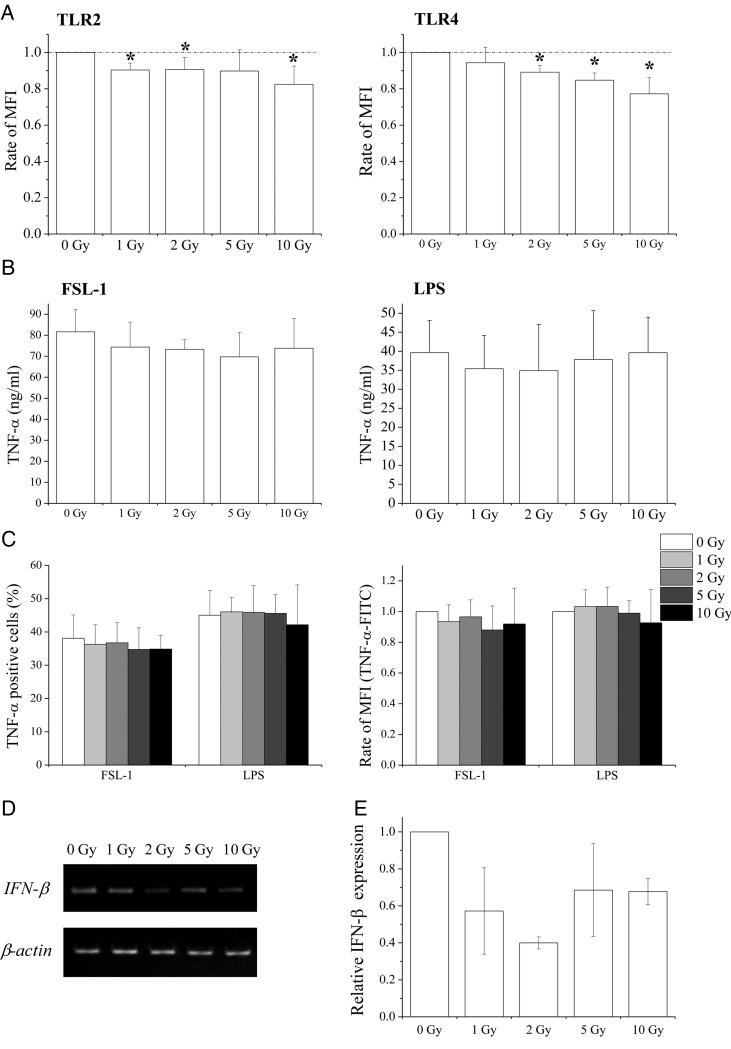
Effects of X-irradiation on TLR2 and TLR4 expression and response to ligands in macrophage-like cells. (**A**) Non- or X-irradiated macrophage-like cells were cultured for 24 h and the expression of TLR2 and TLR4 was analyzed using flow cytometry. Data are presented as the mean ± SD of four independent experiments; single asterisk indicates *P* < 0.05 compared with non-irradiated control. (**B**) Non- or X-irradiated macrophage-like cells were cultured for 24 h, and FSL-1 or LPS were added to culture supernatants. After an additional 24 h of culture, culture supernatants were harvested and TNF-α concentrations were determined using ELISA. Data are presented as the mean ± SD of four independent experiments. (**C**) Non- or X-irradiated macrophage-like cells were cultured for 24 h, were then stimulated with FSL-1 or LPS for 8 h, and the expression of intracellular TNF-α was analyzed. Data are presented as the mean ± SD of four independent experiments. (**D, E**) Non- or X-irradiated macrophage-like cells were cultured for 24 h and LPS was added to culture supernatants. After an additional 24 h of culture, RNA was extracted from cells and the expression of IFN-β was determined using RT-PCR (D) and real-time quantitative RT-PCR (E) analyses. Data from real-time quantitative RT-PCR experiments are presented as the mean ± SD of three independent experiments.

The effects of X-irradiation on the expression of TLR2 and TLR4 in macrophage-like cells were investigated. In contrast with THP1 monocytes, X-irradiation decreased cell surface expression of TLR2 and TLR4 in THP1 macrophage-like cells in a dose-dependent manner (Fig. 3A).

Subsequently, TNF-α production by macrophage-like cells was examined after FSL-1 or LPS stimulation. In these experiments, TNF-α concentrations in culture supernatants from non-stimulated macrophage-like cells were ∼180 pg/ml (data not shown), and were dramatically increased to ng/ml levels after stimulation with FSL-1 or LPS (Fig. 3B), whereas only pg/ml concentrations were observed in THP1 monocytes (Fig. 2C). Although X-irradiation decreased cell surface TLR expression in macrophage-like cells, the production of TNF-α in these cells was comparable with that in non-irradiated cells (Fig. 3B and C).

Because LPS induces both TNF-α and antiviral cytokines (such as IFN-β [6]), the effects of X-irradiation on LPS-induced IFN-β were examined in macrophage-like cells. Although non-stimulated macrophage-like cells did not express IFN-β mRNA (data not shown), IFN-β mRNA expression was observed after LPS stimulation (Fig. 3D) and remained after X-irradiation (Fig. 3D). However, IFN-β expression in X-irradiated macrophage-like cells was lower than that in non-irradiated cells (Fig. 3D and E). IFN-β was then examined in culture supernatants using ELISA, but was not present at detectable levels ( < 50 pg/ml, data not shown).

### Involvement of MAPK in the regulation of TLR expression after X-irradiation

**Fig. 4.**
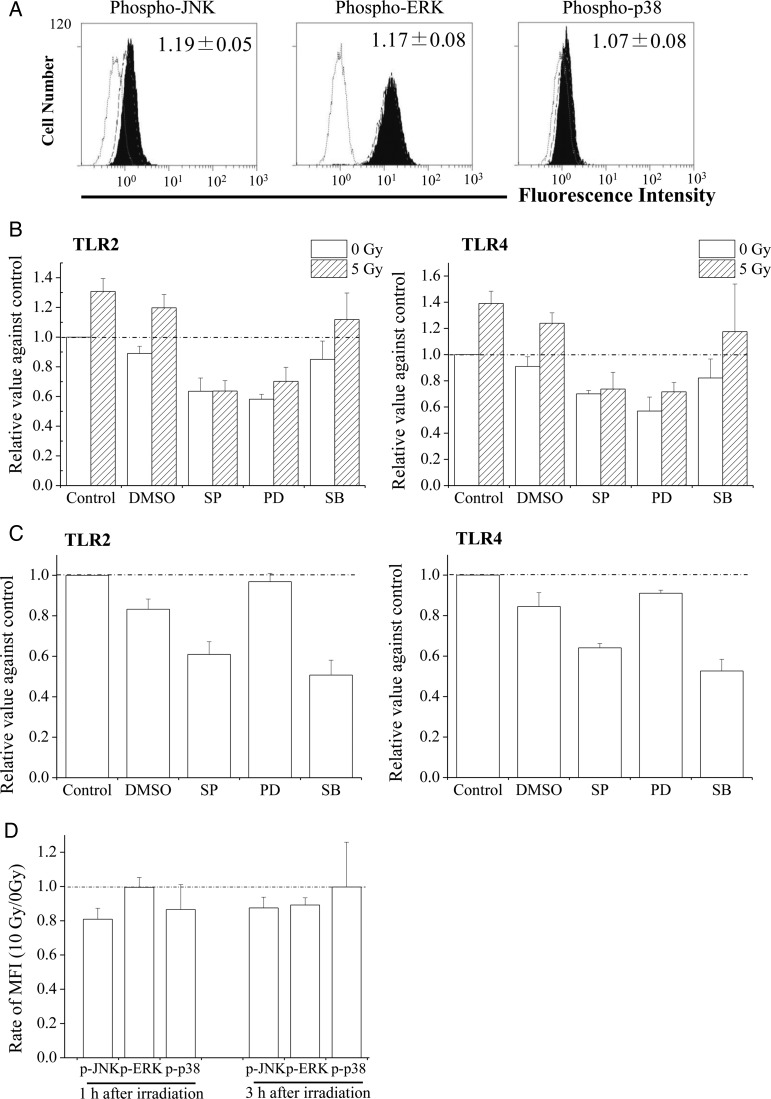
Effects of MAPK on the regulation of TLR2 and TLR4 expression. (**A**) Non- or 5 Gy-irradiated THP1 monocytes were cultured for 1 h, and the expression of phosphorylated MAPK was subsequently analyzed. Filled gray bars indicate MAPK expression in 5 Gy-irradiated THP1 monocytes. Dotted and broken lines indicate isotype and non-irradiated controls, respectively. (**B**) MAPK inhibitors were added to the culture medium 30 min before X-irradiation, and then cells were exposed to 5 Gy. After 24 h of culture, TLR2 and TLR4 expression was analyzed. Inset numbers indicate the relative values of MFI (5 Gy/0 Gy). Data are presented as the mean ± SD of three independent experiments. (**C**) TLR2 and TLR4 expression in macrophage-like cells treated with MAPK inhibitors for 24 h; data are presented as the mean ± SD of three independent experiments. (**D**) Non- or 10 Gy-irradiated macrophage-like cells were cultured for 1 or 3 h, and the expression of phosphorylated MAPK was analyzed. The relative values of MFI (10 Gy/0 Gy) are shown. Data are presented as the mean ± SD of three independent experiments. SP, PD, and SB indicate SP600125 (JNK inhibitor), PD98059 (ERK inhibitor), and SB203580 (p38 inhibitor), respectively.

MAPK signaling pathways are known to regulate TLR expression [17, 18]. Thus, MAPK-mediated regulation of TLR2 and TLR4 expression was investigated after X-irradiation using MAPK inhibitors. Initially, the relationship between MAPK and TLR2 and TLR4 expression was examined in THP1 monocytes. In these experiments, levels of phosphorylated JNK and ERK in 5 Gy-irradiated cells were ∼20% higher than in non-irradiated cells 1 h after X-irradiation (Fig. 4A). Whereas 24-h treatments of non-irradiated THP1 monocytes with the MAPK inhibitors PD98059 (ERK inhibitor) or SP600125 (JNK inhibitor) decreased TLR2 and TLR4 expression, treatment with SB203580 (p38 inhibitor) did not (Fig. 4B). In irradiated cells, treatment with the MAPK inhibitor SP600125 abolished radiation-induced upregulation of TLRs (Fig. 4B), whereas PD98059 only partially suppressed radiation-induced TLRs.

Finally, the involvement of MAPK in TLR2 and TLR4 expression was examined in macrophage-like cells. As shown in Fig. 4C, treatment with SB203580 or SP600125 decreased TLR2 and TLR4 expression, indicating that JNK and p38 are involved in TLR expression in macrophage-like cells. Phosphorylated JNK levels in 10 Gy-irradiated macrophage-like cells were lower than those in non-irradiated cells at 1 h and 3 h after X-irradiation (Fig. 4D). Moreover, phosphorylated p38 levels were slightly lower in 10 Gy-irradiated cells, but only at 1 h after X-irradiation.

## DISCUSSION

The present study demonstrates that X-irradiation affects the expression of TLR2 and TLR4, and that the response to PAMPs depends on the differentiation state of the THP1 monocytes. In THP1 monocytes, X-irradiation increased TLR2 and TLR4 expression (Fig. 2B) and enhanced ligand-induced TNF-α production (Fig. 2C and D); this suggests that X-irradiation enhances the response of THP1 monocytes to PAMPs by upregulating TLRs. However, despite downregulation of TLR expression, production of TNF-α by macrophage-like cells was retained after X-irradiation (Fig. 3A–C). Thus, responses to PAMPs do not necessarily correlate with TLR expression. In a previous report, we showed decreased responses of DCs that were derived from X-irradiated monocytes to LPS compared with non-irradiated cells, despite similar TLR4 expression [13]. In contrast, LPS-inducible IFN-β expression in macrophage-like cells was attenuated by X-irradiation (Fig. 3D and E). After LPS recognition, TLR4 activates adaptor proteins such as myeloid differentiation factor 88 (MyD88) and Toll/IL-1R domain-containing adaptor inducing IFN (TRIF) [19]. The MyD88 signaling pathway stimulates production of pro-inflammatory cytokines such as TNF-α, whereas TRIF signaling leads to the production of antiviral cytokines such as IFN-β. Therefore, it is possible that X-irradiation affects TRIF-dependent (but not MyD88-dependent) signaling pathways in macrophage-like cells.

Interestingly, the regulation of TLR2 and TLR4 expression by MAPK varied depending on the cell differentiation state. In brief, JNK and ERK are involved in TLR expression on THP1 monocytes (Fig. 4B), whereas JNK and p38 are involved in macrophage-like cells (Fig. 4C). In agreement with previous reports [20, 21], phosphorylation of JNK was increased in THP1 monocytes after X-irradiation (Fig. 4A). However, X-irradiation decreased the levels of phosphorylated JNK in macrophage-like cells. These results suggest that X-irradiation regulates TLRs through JNK, depending on the cell differentiation state. It remains unclear why JNK activation by ionizing radiation depends on the cell differentiation state. However, given that JNK is sensitive to intracellular redox signaling [22], and antioxidant enzymes such as superoxide dismutase 2 are involved in radiation resistance [23], it is possible that cell differentiation by PMA promotes antioxidant systems, which leads to the differences in both radiation sensitivity and JNK activation after exposure to ionizing radiation (Fig. 1B and C and Fig. 4A and D).

Production of TNF-α in X-irradiated THP1 monocytes was higher than in non-irradiated cells after stimulation with PAMPs (Fig. 2C and D). TLRs recognize both PAMPs and damage-associated molecular patterns (DAMPs), such as heat shock proteins and high mobility group box 1, which are released from injured tissues and dead cells [24]. Lambros *et al*. reported upregulation of several DAMP genes in tissues exposed to ionizing radiation [25]. Furthermore, death of cancer cells by medical therapy causes the release of DAMPs [26]. Therefore, it is possible that ionizing radiation induces inflammation through the release of DAMPs and through the upregulation of TLRs in irradiated immune cells even in the absence of PAMPs. To clarify the involvement of TLRs in radiation-induced inflammation, further studies of the effects of radiation on other TLR-expressing cell types such as endothelial cells [27] are required.

## CONCLUSION

In conclusion, the present data demonstrate that ionizing radiation affects TLR expression by activating JNK and PAMP responses, depending on the differentiation state of cells. These insights into the regulation of TLRs by ionizing radiation elucidate the mechanisms of radiation-induced impairment of immune function and radiation-induced inflammation.

## FUNDING

This work was supported by a Japan Society for the Promotion of Science (JSPS) KAKENHI Grant-in-Aid for Young Scientists (B; No. 23791383 and No. 25861053). This study was also partially supported by a Hirosaki University Grant for Exploratory Research by Young Scientists (2013) and a Priority Research Grant for Young Scientists Designated by the President of Hirosaki University. Funding to pay the Open Access publication charges for this article was provided by JSPS KAKENHI Grant-in-Aid for Young Scientists (B;No. 25861053).
